# Seasonal optical backscattering in hypersaline waters: In-situ observations and data analysis

**DOI:** 10.1371/journal.pone.0314567

**Published:** 2025-03-07

**Authors:** Arwa Najah, Maryam R. Al Shehhi

**Affiliations:** Civil and Environmental Engineering, Khalifa University of Science and Technology, Abu Dhabi, United Arab Emirates; Central Marine Fisheries Research Institute, INDIA

## Abstract

Relationships of backscattering coefficients with the physical and biological properties in hypersaline waters like the Arabian Gulf are poorly studied. They may differ from other non-hypersaline waters which contribute the majority of data used to develop and parameterize optical models. Herein, we analyze the covariation of salinity, temperature, Chlorophyll-a (Chl-*a*), color dissolved organic matter (CDOM), ammonium, and turbidity with seawater backscattering coefficients b_b_(λ). This analysis is based on *in-situ* measurement of total backscattering and satellite total backscattering coefficients retrieved from the Visible Infrared Imaging Radiometer Suite (VIIRS). The *in-situ* measurements have been collected in the southern region of the Arabian Gulf waters, characterized by salinity and high evaporation rate. The results showed that turbidity is the main contributor to the increase in b_b_ (λ) which could reach up to 77%. In addition, an increase in salinity is associated with an increase in the b_b_ (λ) up to 19% especially at 532 nm. As for the temperature, we found that b_b_(λ) during the winter season is higher than in the summer season which could be due to the mixed effect of the surface sedimentation and the well-mixed column during winter. As for the Chl-*a*, there is a noticeable covariation between b_b_ (λ) and the Chl-*a* concentration. Thus, we examined the probability distribution of Chl-*a* against different ranges of b_b_(λ) and found that Chl-*a* can follow log-normal and Weibull probability distribution which can be used for different b_b_(λ) ranges of 532 and 488 nm. Based on this study, we found that the hypersaline waters of the Gulf have b_b_ scattering patterns that are consistent with the previously reported studies elsewhere.

## 1. Introduction

Spectral backscattering is a critical parameter in ocean optics, directly influencing the Bidirectional Reflectance Distribution Function (BRDF) and governing the relationship between downwelling and upwelling radiance [1]. Its magnitude plays a vital role in remote sensing algorithms, particularly those independent of reflectance ratios or water-leaving radiance [2,3]. Thus, backscattering is indispensable for both passive and active remote sensing applications, such as satellite-based ocean monitoring and in-situ observational tools. Furthermore, backscattering serves as a proxy for biogeochemical cycling, offering insights into the concentrations of key oceanographic parameters such as chlorophyll-a (Chl-*a*), particulate organic carbon (POC), and total suspended matter (TSM) [4]. Therefore, accurate measurements of backscattering are vital for interpreting water reflectance signals and retrieving critical biogeochemical parameters needed for monitoring phytoplankton blooms, water quality, sediment transport, and river discharge in coastal oceans [5]. Given its utility across multiple oceanographic disciplines, the accurate quantification of backscattered light is essential for studying particle dynamics, biogeochemical processes, and overall ocean health.

The origin of backscattering in marine environments is predominantly due to two factors: particle scattering and molecular scattering from pure seawater. This relationship is commonly expressed as the sum of individual backscattering coefficients for water and particles:


$$b_{b}\left(\lambda \right)=b_{bw}\left(\lambda \right)+b_{bp}\left(\lambda \right)$$ (1)

where $b_{bw}\left(\lambda \right)$refers to molecular scattering from water, and $b_{bp}\left(\lambda \right)$ corresponds to particulate scattering. Theoretical studies using Mie scattering have shown that backscattering correlates strongly with particle mass concentration, although less strongly with other particle measures like POC and Chl-*a* [6].

In coastal waters, particle backscattering typically dominates and significantly influences the propagation of light [7], determining the magnitude of surface reflectance [8]. While in clearer oceanic waters, molecular scattering becomes more prominent. The particle backscattering coefficient, for example, can increase by six orders of magnitude from relatively clear to highly turbid waters. Despite this understanding, applying theoretical models to real-world scenarios presents challenges due to variability in particle size, shape, composition, and refractive index [9]. Moreover, the optical properties of seawater are influenced by dissolved organic matter, suspended particles, and gas bubbles, all of which contribute to scattering [10,11]. Variability in the particle backscattering coefficient is largely driven by changes in these factors. Most of the current understanding of how backscattering interacts with particle characteristics stems from modeling studies that use idealized particle assemblages along with theoretical descriptions of particle absorption and scattering [12].

Existing models of backscattering, derived largely from open ocean waters [13], may not fully capture the unique optical characteristics of coastal and hypersaline environments like the Arabian Gulf. The Arabian Gulf, characterized by extreme evaporation rates, high salinity, and significant anthropogenic activities, presents a complex optical regime [14,15]. These factors, coupled with seasonal phytoplankton blooms and heavy sedimentation, make it a particularly challenging area for applying generalized backscattering models developed for less extreme environments. A critical issue arises when considering the contribution of bio-optical properties to backscattering in such environments. Although many studies have established a relationship between particle backscattering and Chl-*a* concentration in open oceans, these relationships may not hold in hypersaline environments like the Arabian Gulf [16,17]. This highlights the need for region-specific studies to understand how physical, chemical, and biological properties influence backscattering.

The current study focuses on addressing the knowledge gap surrounding backscattering variability in the Arabian Gulf. The primary objective is to analyze how changes in Chl-*a*, salinity, temperature, and sediments influence backscattering coefficients in this hypersaline context. Existing studies have explored linear or non-linear relationships between backscattering and bio-optical properties, but this research provides a critical examination of these dynamics in the unique conditions of the Arabian Gulf. By integrating satellite data and in-situ observations collected during the winter and summer of 2013, 2014, and 2016 along the southern Gulf’s coastline, the study offers a comprehensive analysis of the physical and biological factors driving backscattering. The findings are expected to refine optical models, improve the understanding of biogeochemical processes, and support environmental management efforts. Given the region’s environmental and economic importance, these insights are essential for advancing scientific knowledge and guiding sustainable management of marine resources, while mitigating anthropogenic impacts on the Gulf’s coastal ecosystems.

## 2. Data and methods

### a. Field campaign

**Fig 1:**
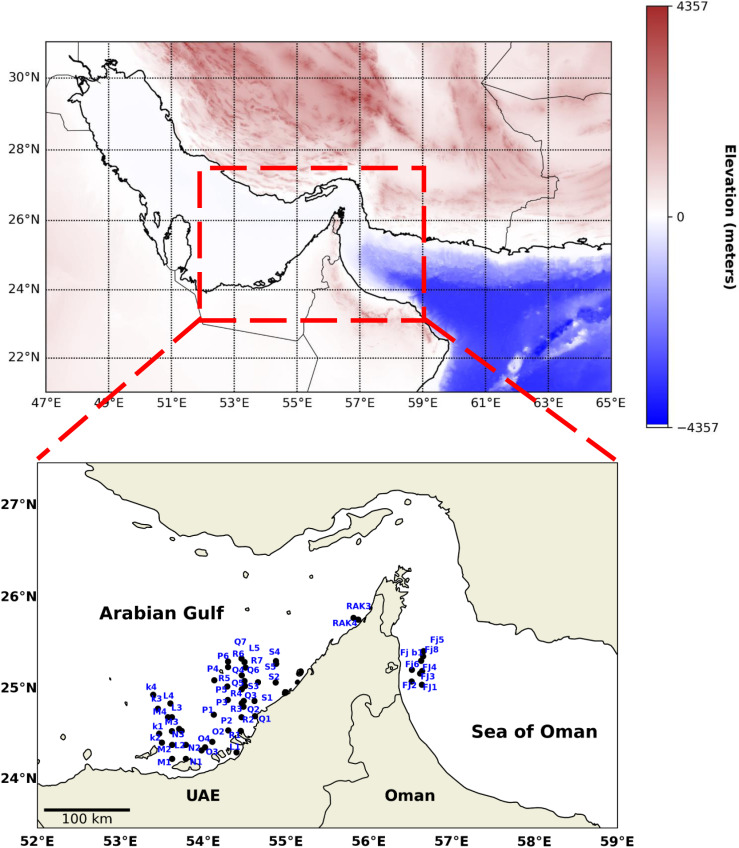
The map of the studied area in the southern region of the Arabian Gulf and the western part of the Sea of Oman. The stations show the coordinates of the field measurements along several transects. Topographic elevation map from GEBCO Compilation Group (https://www.gebco.net/data_and_products/gridded_bathymetry_data/).

In-situ measurements were conducted between June 26, 2013, and March 29, 2016, covering both the western coast of the Arabian Gulf and the eastern coast of the Sea of Oman. The measurements were collected at 54 stations (as shown in Fig 1) along 13 transects. Of these, 11 transects ran parallel to the Abu Dhabi beach, 1 along the Ras Al Khaimah shoreline, and 1 along the Fujairah coastline in the Gulf of Oman. Sampling stations along each transect ranged from one to seven, depending on the transect’s length and geographical position. Sampling started from the point nearest to the shoreline and extended outward to additional sites, maintaining a consistent distance between adjacent stations as specified by the established grid system. This consistent spacing ensured comprehensive coverage of the study area and allowed for precise spatial analysis.

Coastal areas in the Arabian Gulf are critical zones where land-sea interactions, like runoff and human activities, have the most impact on water quality. By focusing on these dynamic regions, we capture the most significant changes in optical properties. Additionally, the shallow, semi-enclosed nature of the Arabian Gulf means coastal processes influence the entire water body. Currents mix coastal and offshore waters, making nearshore measurements representative of broader trends. Therefore, the chosen station distribution effectively captures the key patterns in the study area. For more details on the hydrography and the physics of the region, see [18].

Meteorological conditions and seawater quality were measured at each station using standardized instruments. A Thermo-Anemometer was used to capture meteorological parameters such as air temperature and wind speed. The water depth at each site was monitored and recorded using the ship’s control screen, while water quality parameters like sea surface temperature, pH, ammonium, and salinity were measured using a portable meter. To estimate water turbidity, which is indicative of sediment levels, Secchi disk depth (SDD) measurements were taken. Water samples were also collected using Niskin bottles and transported to the laboratory for further analysis, specifically to extract Chl-*a* concentrations as described in reference [19].

In addition to water quality and meteorological data, backscattering coefficients at four distinct wavelengths (440 nm, 488 nm, 532 nm, and 650 nm), as well as fluorescence measurements for Chl-*a* and colored dissolved organic matter (CDOM), were obtained using the WETLabs ECO-triplet BBFL2B. The ECO-triplet sensor was deployed to a depth of 2 meters at each of the stations. This instrument emits light at a specific wavelength and detects fluorescence, reporting the data in grams per liter (g/l) for Chl-*a* and parts per billion (ppb) for other fluorescence measurements. Additionally, the ECO-triplet records the backscattering of the medium, expressed as volume scattering coefficients with units of m ⁻ ¹ sr ⁻ ¹. The sensor outputs raw data as digital values (DVs), which range between 0 and 4120 ± 5 counts. These raw DVs were then converted into backscattering coefficients (b_b_) using calibration formulas provided in the sensor’s manual.

Data were collected across different seasons to capture variability throughout the year. To maintain measurement precision, all instruments were calibrated regularly. Additionally, both raw and processed data were securely stored and backed up. Consequently, this detailed procedure ensured accuracy in the measurements and consistency across all stations, thereby providing reliable analysis of the collected data.

### b. Surface data interpolation and satellite data

The rationale behind the modeling system in this study is to create a comprehensive spatial representation of the variability in physical, chemical, and optical properties of the marine environment. To achieve this, the collected in-situ data were horizontally interpolated to estimate values at locations between sampled points. This approach is essential for understanding the spatial distribution of backscattering coefficients and other environmental parameters across the study area, utilizing the Inverse Distance Weighting (IDW) interpolation method. IDW is a deterministic approach that estimates unknown values based on the weighted average of known data points, with weights decreasing with distance (Eq. 2). This method effectively provides a smooth spatial representation by giving more influence to data points closer to the location of interest. 


$$Z_{j}=\frac{\sum_{i}\frac{Z_{i}}{d^{n}{}_{ij}}}{\sum_{i}\frac{1}{d^{n}{}_{ij}}}$$ (2)

where $Z_{j}$ is the interpolated value, $Z_{i}$ is the value of the known point, $d_{ij}$ is the distance between these two points, and n is a user-selected exponent (often 1, 2, or 3) which, determines the degree to which the nearer point(s) are preferred over more distant points. This approach ensures that the generated maps provide a comprehensive view of the study area.

To complement the in-situ measurements and enhance the spatial maps, satellite-derived data were incorporated. Specifically, VIIRS Level-2 products for backscattering coefficients (b_b_(λ)) were obtained at the same wavelengths as the *in-situ* measurements. They were retrieved for the winter and summer seasons from the ocean color website. This satellite data supports and validates the findings from the field measurements, providing an additional layer of spatial context and temporal coverage.

By combining in-situ measurements with satellite data, the study aims to achieve a detailed understanding of how changes in environmental factors such as Chl-*a* concentrations, sediment levels, temperature, and salinity affect backscattering coefficients. This methodology ensures that the spatial variability of these properties is accurately represented, facilitating a comprehensive analysis of their impact on marine environments.

## 3. Results and discussion

### 3.1 Spatial-temporal variability of physical and biochemical properties

**Fig 2:**
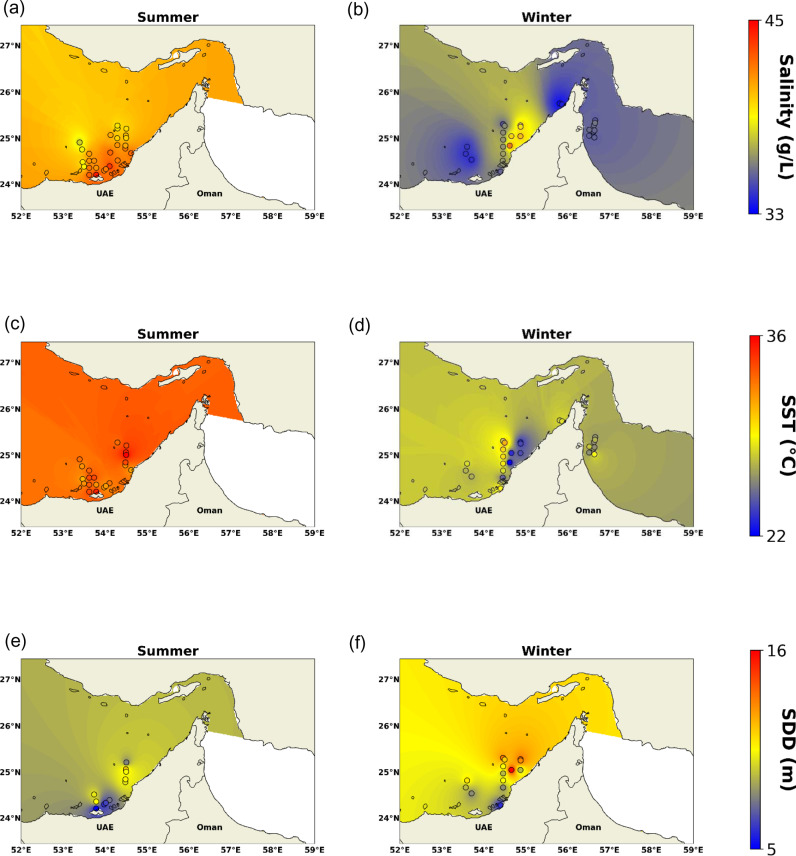
Surface maps generated by IDW of (a-b): salinity, (c-d): SST, and (e-f): SDD during the summer and winter seasons.

The salinity measurements in the study area have shown relatively high ranges, especially during summer ranging from 33 to 45 g/L (g kg^ − 1^), with the highest values found in June (summer: Fig 2a) and the lowest value in November (winter: Fig 2b). The salinity has also shown a trend of decreasing from nearshore to offshore. This pattern is linked to higher evaporation and weaker circulation in nearshore waters than in offshore waters, and Arabian Gulf circulation conditions are characterized by the outflow of highly saline water into the Gulf of Oman and the inflow of fresh water into the Gulf [20,21]. As for sea surface temperature (SST), the values have ranged between 22 and 36 °C. Summer had higher values (>30 °C) throughout the whole study area (Fig 2c). Whereas winter had relatively lower values which could reach 22 °C, where the lowest was in the middle of the Gulf (Fig 2d). Finally, the values of SDD varied between 5 and 16 m with low values in coastal waters while high values in offshore waters and winter. The highest SDD was found in March (winter) and the lowest SDD was found in June (summer) as shown in Fig 2e, 2f.

**Fig 3:**
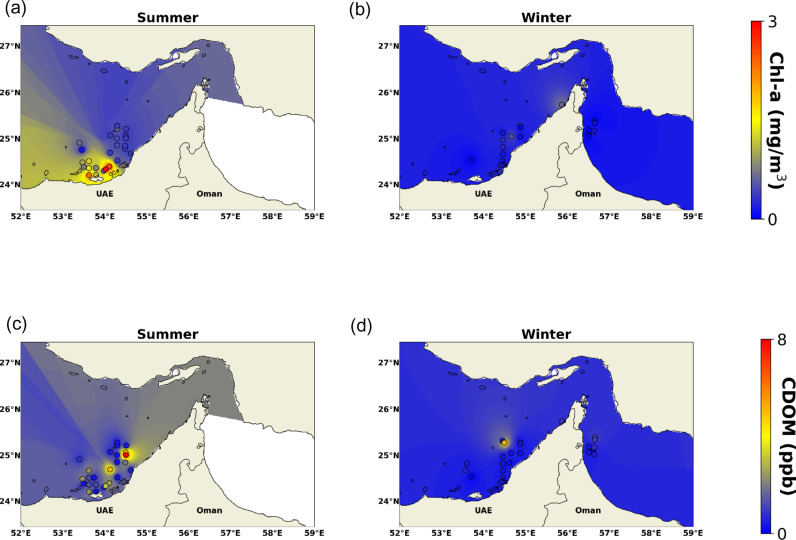
Surface maps generated by IDW of (a-b): Chl-*a*, and (c-d): CDOM during the summer and winter seasons.

Regarding the Chl-*a* concentration, it varied between 0 and 3 mg m^ − 3^ and was generally low (0.5–1 mg m^ − 3^) during non-bloom conditions. High Chl-*a* was recorded during summer (Fig 3a) in the southwestern part of the region, whereas low Chl-*a* concentrations were detected during winter (Fig 3b). Similarly, for CDOM, winter had mostly low values (<1 ppb) whereas higher values were observed in summer (>5 ppb, Fig 3c). CDOM decreased overall in winter (Fig 3d) due to lower biological activity and a drop in terrestrial input from river run-off [22]. As for the ammonium concentrations, they varied significantly between summer and winter. The highest value was 94 mg/L in November, whereas the lowest values were at the end of the summer season (October). Summer’s low ammonium levels can be linked to algae’s high photosynthesis, which eliminates ammonium. Algae take up little ammonia during the winter, but the ammonia supply remains, and it is controlled by the total amount of feed provided to the water during the previous growing season [23].

### 3.2 Spatial variability of b _b_ (λ)

**Fig 4:**
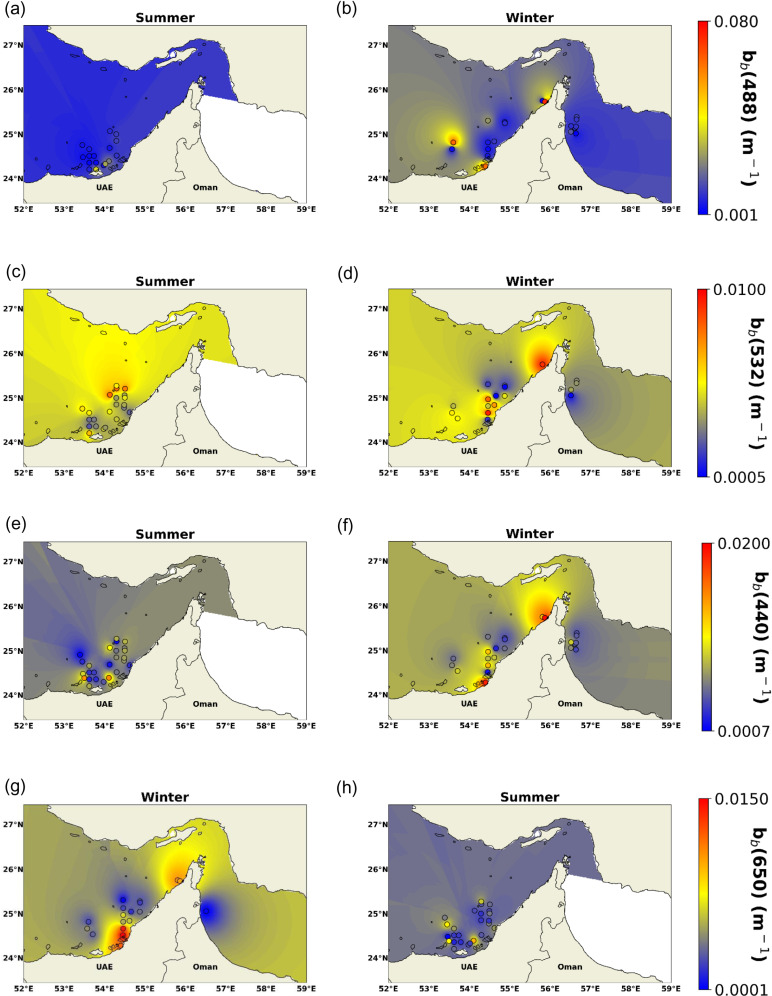
Surface maps generated by IDW of (a-b): b_b_ (488), (c-d): b_b_ (532), (e-f): b_b_ (440), and (g-h): b_b_ (650).

b_b_(λ) has shown high spatial variability in winter and summer seasons. In terms of seasonal changes, b_b_(λ) values are commonly higher (can exceed 0.008 m^-1^) in the winter season compared to summer (~avg: 0.005 m^-1^). The coastal areas don’t show higher b_b_(λ) values compared to the offshore regions except for a few stations where the productivity is higher thus increasing b_b_(λ) (~0.005 m^-1^). In addition, b_b_(λ) is found to be lower (~avg:0.0058 m^-1^) in the eastern region along Sea of Oman compared to the inner Arabian Gulf region with b_b_(λ) values of (~avg:0.007 m^-1^).

b_b_ is also found to be changed as per the wavelength. For example, b_b_(488) showed the highest values compared to the other three wavelengths due to the effect of sediments on this spectral range. In fact, remote sensing reflectance at 488 nm is commonly used to determine the attenuation coefficient which is significantly dependent on sediment levels. Hence, b_b_(488) during winter is found to be higher in the turbid southern part of the Arabian Gulf and the northern part toward the Strait of Hormuz with values exceeding 0.05 m^-1^. In addition, b_b_(488) is found to be lower along the coast of the Sea of Oman due to the lower sediments in this region. b_b_(488) was also affected by the season, as b_b_(488) doesn't exceed 0.035 m^-1^ during summer (Fig 4a), compared to winter where b_b_(488) could reach up to 0.08 m^-1^ (Fig 4b).

Similarly, b_b_(532) showed high spatial variability ranging from 0.0005 to 0.01 m^-1^. High b_b_(532) values were observed in the southern Arabian Gulf and near the strait of Hormuz. However, it is interesting to note that b_b_(532) showed higher values during the summer season (Fig 4c) compared to winter (Fig 4d). On the other hand, b_b_(532) and b_b_(650) showed the lowest values compared to the other two wavelengths, b_b_(488) and b_b_(440). Moreover, both b_b_(440) and b_b_(650) showed similar behavior of higher values during winter (Fig 4f, 4h) and lower during summer (Fig 4e, 4g). However, b_b_(650) values showed a significant seasonal change compared to b_b_(440). In addition, b_b_(650) values were found to be higher (>0.01 m^-1^) where the productivity is higher due to the scattering caused by phytoplankton species. Overall, high backscattering was observed in the blue region and decreased towards longer wavelengths; the same pattern was observed in coastal and turbid waters of the Mediterranean Sea, Lake Taihu in China, Gulf of Mexico, and other places [3,24–26].

**Fig 5:**
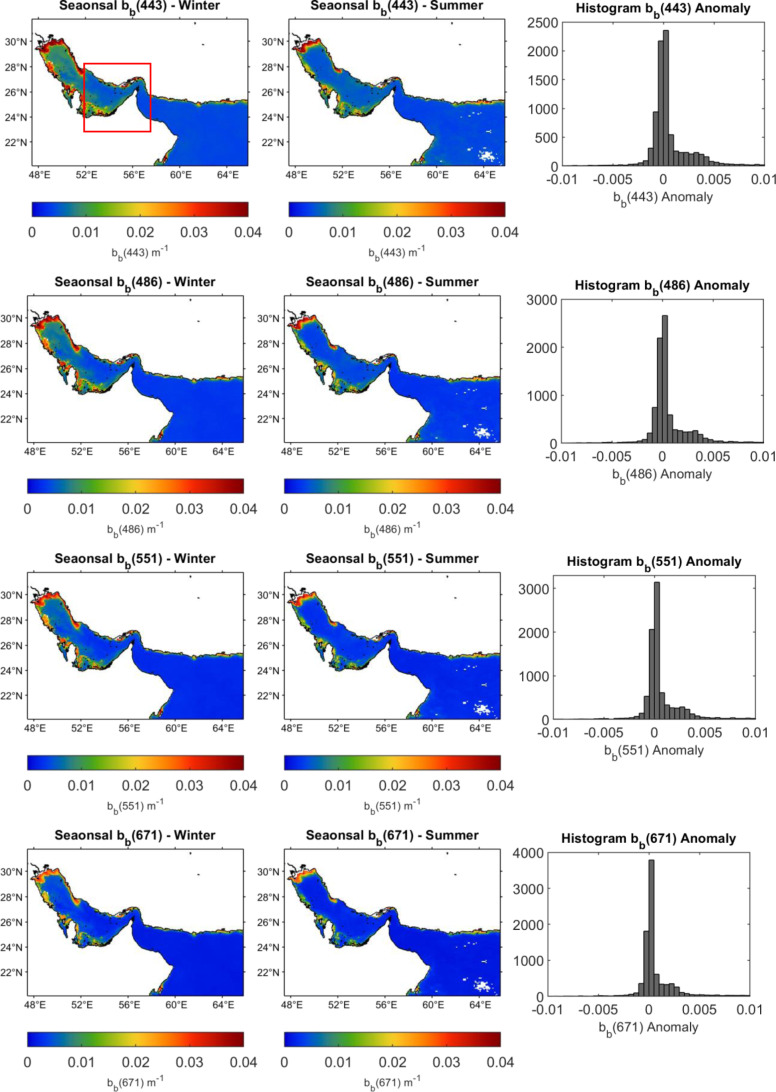
Surface seasonal variations of b_b_(λ) at 443, 486, 551 and 671 nm over the study area during summer and winter. The histograms show the anomalies between b_b_ (λ) winter and summer. The maps are created by MATLAB R2020a www.mathworks.com.

The high surface values of b_b_(λ) had also been observed using VIIRS season maps as shown in Fig 5. It is obvious that during the winter season higher surface values were observed especially over the northern region of the Arabian Gulf compared to the summer season. This is explained by that during winter most of the northern part of the Gulf becomes vertically mixed, with maximum density and highest salinity off the Saudi coast [27]. Therefore, the total column showed similar levels of suspended matter from the bottom given the shallow depth of the basin. However, in the southern region around the same geo-boundaries of Fig 5, the difference between summer and winter was less notable which could be due to the effect of the inflow coming from the Sea of Oman affecting the mixing of this region thus the b_b_(λ) get also affected by the physical and biological conditions. This tendency is observed for the four wavelengths in Fig 5 where it was also obvious from the histograms, that the anomalies showed mostly positive values suggesting an increase in b_b_(λ) during winter compared to summer over all wavelengths.

### 3.3 Vertical profiles of b_b_(λ)

In addition to the horizontal variability of b_b_(λ), the vertical behavior of light backscattering can also co-vary with the water’s physical and biochemical properties. Therefore, based on the *in-situ* b_b_(λ) collected in the Gulf and Sea of Oman water, the vertical profiles of b_b_(λ) at three wavelengths 440, 532 and 650 nm had been analyzed in terms of its vertical variability as well as its dependency on the physical properties (SST, salinity & turbidity) and the biochemical properties (Chl-*a*, Ammonium, & CDOM). The vertic*a*l profiles of b_b_(488) have been excluded in the analysis here due to the noise in the data making it unreliable. Due to the high variability of the vertical b_b_(λ), these vertical profiles are presented as boxplots for every 0.1 m of depth from the water surface down to 1.6 m collected at different stations.

#### 3.3.1 Variation of salinity versus b_b_(λ).

**Fig 6:**
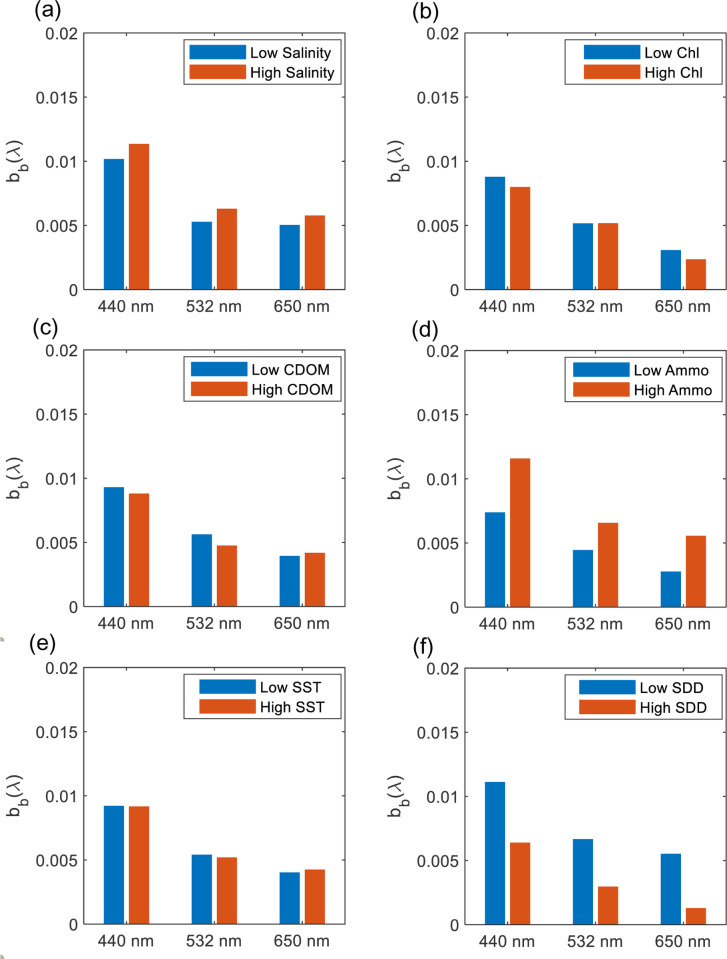
The depth averaged b_b_ (λ) for different levels of high and low: (a) salinity, (b) Chl-*a*, (c) CDOM, (d) Ammonium, (e) SST and (f) SDD.

**Fig 7:**
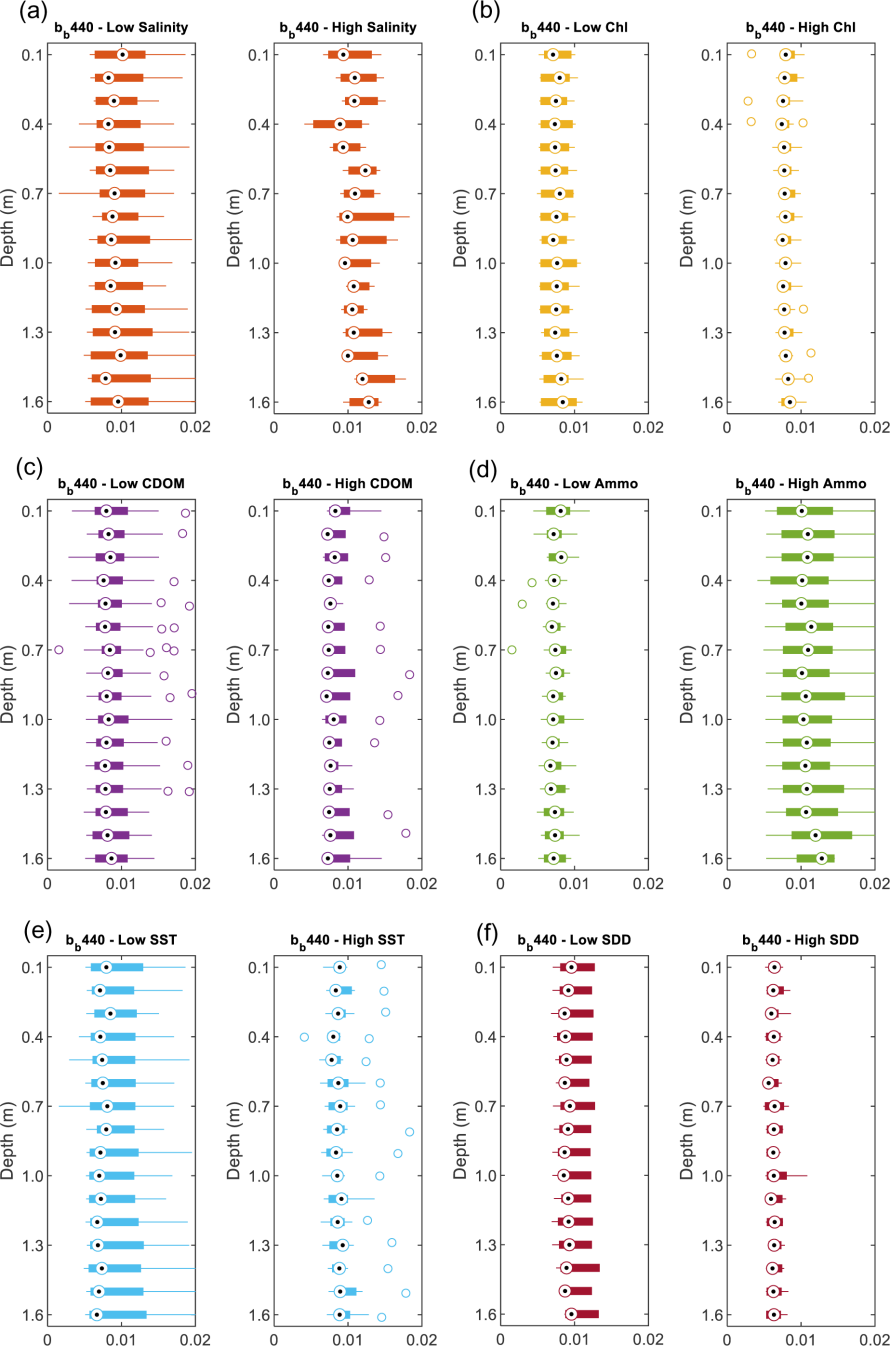
The vertical profile of b_b_ (440) for different levels of high and low: (a) salinity, (b) Chl-*a*, (c) CDOM, (d) Ammonium, (e) SST and (f) SDD.

**Fig 8:**
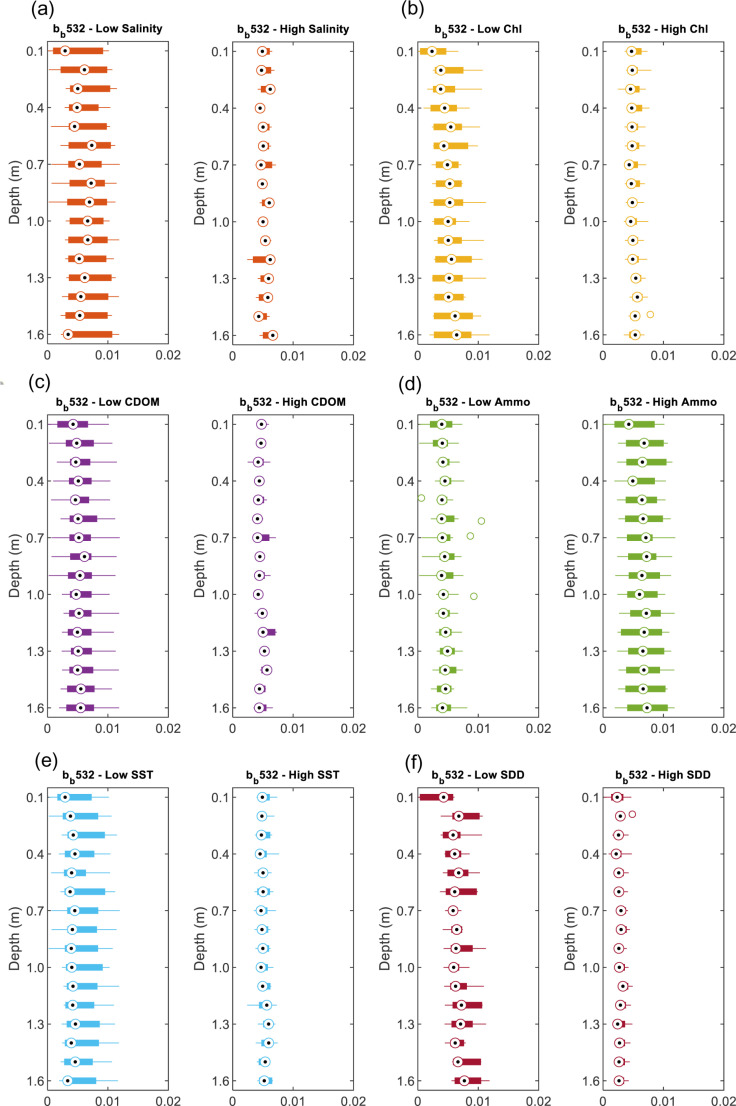
The vertical profile of b_b_ (532) for different levels of high and low: (a) salinity, (b) Chl-*a*, (c) CDOM, (d) Ammonium, (e) SST and (f) SDD.

The variation of the *in-situ* measurement of the total backscattering coefficient and its possibility of changing is examined here for two salinity S levels, low for S: 33 −  38 g/L (g kg^ − 1^) and high for S: 38−45 g/L (g kg^ − 1^). We found that average vertical backscattering increases at wavelengths 440 nm, 532 nm, and 650 nm with increasing salinity, with the maximum increase found at wavelength 532 nm (19%) as shown in Fig 6a. For example, at low S, b_b_ (532) is 0.005 m^−1^ and at high S, b_b_(532) is 0.006 m^−1^ (Fig 8a). The increase in b_b_(λ) has varied between 12% to 19% (Fig 6a and Fig 7a, 8a, 9a). This increase in scattering is consistent with the analyses conducted by Morel where scattering has increased by 30% for the spectral range of 366 - 578 nm at S = 38.4 g kg^ − 1^ compared to the pure water [11]. Similarly, the Zhang model suggested that the total backscattering of the water can deviate by 25% at 443 and 547 nm with changing the salinity and temperature of natural waters (−2 ≤  T ≤  40°C and 0 ≤  S ≤ 40 g kg − 1) [28]. This is covering the shorter wavelength, whereas in our analysis, we have also found the possibility of increasing the backscattering at the longer wavelength (i.e., 650 nm). The increase in salinity has been clearly explained in [29] in which the presence of sea salts induces additional fluctuations in the concentration leading to additional scattering. It is estimated that up to 80% of total backscattering occurs in clear waters due to the nearly isotropic characteristics of the angular distribution of seawater [29,30]. However, this could include uncertainty due to depolarization ratio in which scattering can be enhanced by electrostatic field and reduced because ions are isotropic [31]. But in the case of the hypersaline water, a clear relationship is observed between the salinity levels and the wavelengths. In addition, based on the vertical profiles of backscattering, there is no significant change in the vertical profile for the top one and half meters of the seawater in which backscattering fluctuates over an average value. However, in shallow coastal areas with high salinity, b_b_(440) varies more with depth due to increased sediment resuspension, higher particle concentrations, and the effects of elevated salinity. Enhanced evaporation leads to denser saline layers that accumulate particles, while vertical mixing and bottom reflectance further contribute to the variability in b_b_ (440).

**Fig 9:**
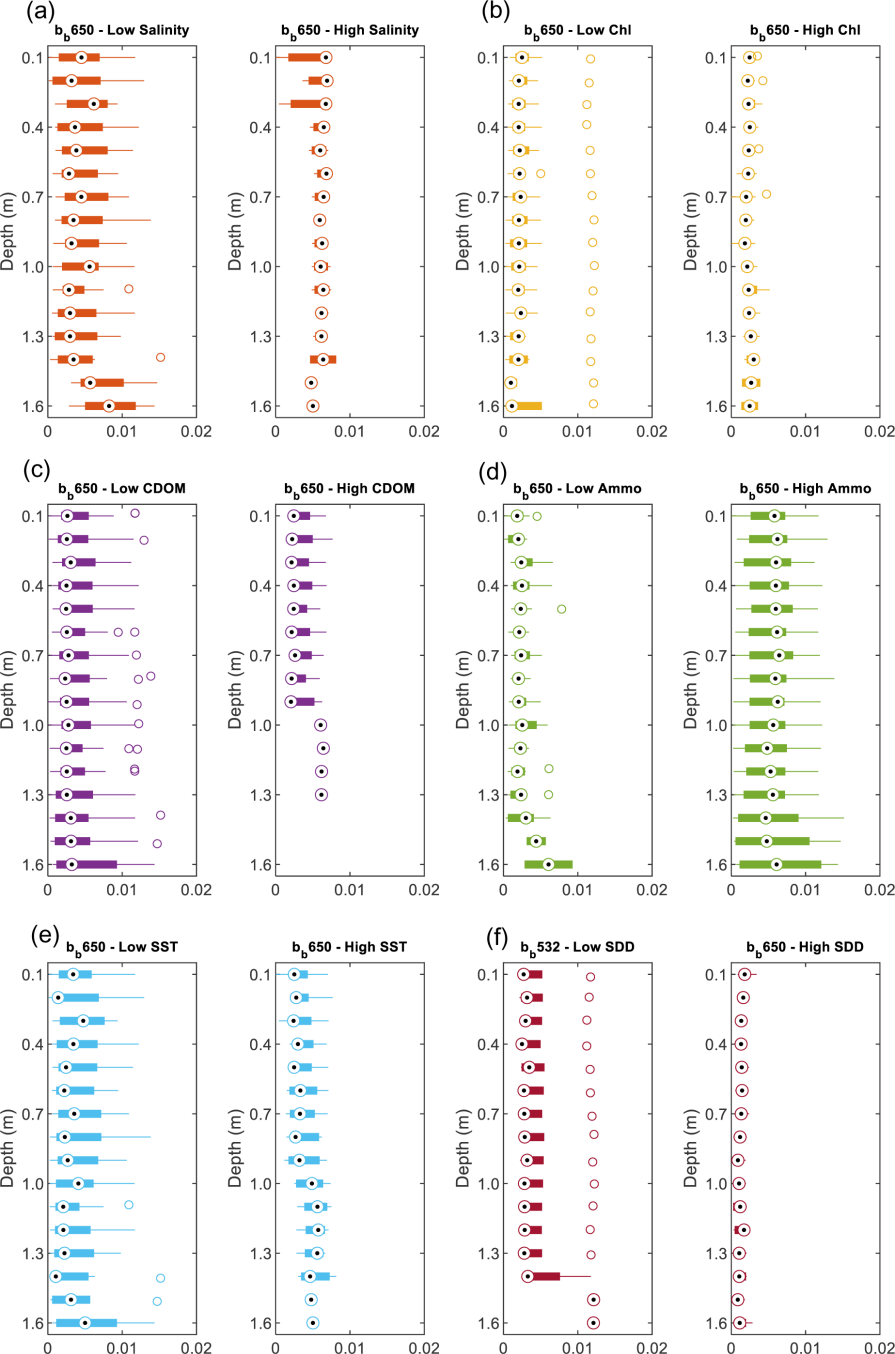
The vertical profile of b_b_ (650) for different levels of high and low: (a) salinity, (b) Chl-*a*, (c) CDOM, (d) Ammonium, (e) SST and (f) SDD.

#### 3.3.2 Variations of Chl*-a*, CDOM, and ammonium versus b_b_(λ).

We found a significant relationship between b_b_ (440) and Chl-*a* in which high Chl-*a* (0.6–3 mg m^ − 3^) has shown lower b_b_(440) values by 9% compared to the low Chl-*a* (0.01 −  0.6 mg mg^ − 3^) as shown in Fig 6b and Fig 7b, 8b, 9b. This relationship could be used to estimate surface Chl-*a* from data collected by *in-situ* autonomous platforms and from remote sensing of ocean color. Although the study area is considered to be *case II* waters in which suspended particles dominate the scattering, our data showed that the strategy of parameterizing the scattering coefficients b_b_ as a function of Chl-*a* could be possible. Although this finding is consistent with the finding of Martinez‐Vicente, V., et al. (2013) in their analysis of the Atlantic oceanic waters where Chl-*a* concentrations less than 0.4 mg m^ − 3^ have shown an effect on particulate backscattering at 470 nm. The resultant effect is found to be opposite to our finding as less Chl-*a* has shown lower b_bp_(470) than 0.003 m^−1^ [32]. This could be explained by the different nature of the Arabian Gulf water as Case II compared to the Atlantic waters. In addition, our results are found to be more consistent with the typical behavior of Chl-*a* as more absorption happens (i.e., not scattering) at the blue (440 nm) and the red wavelength (650 nm) ranges when increasing Chl-*a* concentrations [33]. However, for the other wavelength (532 nm), there is no significant changes been observed.

**Fig 10:**
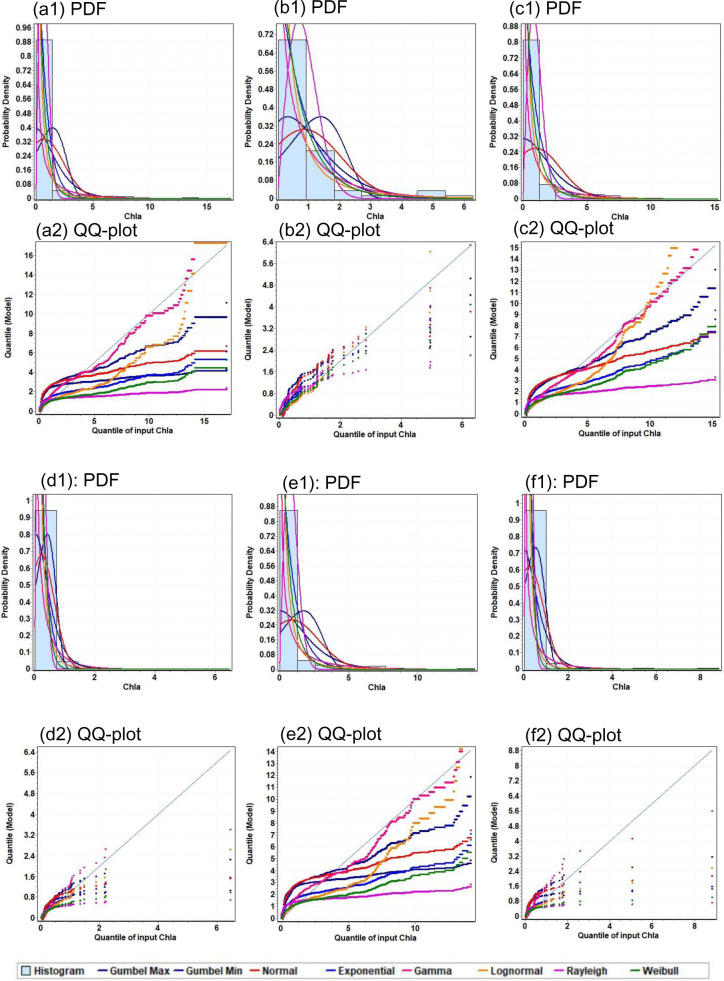
Chl-a probability density function and Q-Q plot for: (a) b_b_ (488) <  0.06, (b) b_b_ (488) >  0.06, (c) b_b_ (532) <  0.01, (d) b_b_ (532) >  0.01, (e) b_b_ (532)/b_b_ (488) < 1 and (f) b_b_ (532)/b_b_ (488) >  1.

Similar to the reported results, there is however a large spread in this relationship between Chl-*a* and b_b_ (λ) makes Chl-*a* a poor predictor of b_b_ [34]. This could be explained by the presence of many cellular- and population-level processes that can affect the relationship [35]. Moreover, phytoplankton groups and community structures can have divergent compositions, which can affect relationships between Chl-*a* and backscattering [34]. However, in order to take advantage of the relation of Chl-*a* with the surface b_b_(488) and b_b_(532) at the surface water, multiple probability distributions were applied to model Chl-*a* under various b_b_ ranges. Based on the Kolmogorov-Smirnov (KS) test, Lognormal and Weibull provided the best fit to the Chl-*a* under all b_b_ ranges as shown in Fig 10. The probability density functions (pdfs) are used here to describe Chl-*a* distributions. Chl-*a* values were selected for multiple conditions of b_b_ (b_b_(488) <  0.06, b_b_(488) >  0.06, b_b_(532) <  0.01, b_b_(532) > 0.01, b_b_(532)/b_b_(488) <  1 and b_b_(532)/b_b_(488) > 1). Fig 10 illustrates the frequency histograms, pdfs, and Q-Q plots for Chl-*a* of each of these conditions. Results indicated that most of the distributions underestimated Chl-*a* values especially for higher values ( > 5 mg m − 3). Based on the parameters of the Lognormal and Weibull pdfs, there are significant variabilities can be observed over the different conditions of b_b_ making this a possibility to predict the probability of Chl-*a* based on b_b_. Table 1 showed the estimated parameters of both Lognormal and Weibull pdfs for the six conditions.

**Table 1:** The estimated parameters of the probability density functions (pdfs) for the lognormal and Weibull distributions. These distributions are estimated for six categories/conditions of b_b_ (488) and b_b_ (532).

Probability Distibution	Lognormal		Weibull	
Parameter	µ	σ	A	B
b_b_ (488) < 0.06	−1.4599	1.3336	0.4727	0.6585
b_b_ (488) > 0.06	−0.8163	1.2846	0.8334	0.8259
b_b_ (532) < 0.01	−1.169	1.4824	0.6726	0.6442
b_b_ (532) > 0.01	−2.0006	0.9898	0.2245	0.9216
b_b_ (532)/b_b_ (488) < 1	−1.2814	1.4022	0.5826	0.6476
b_b_ (532)/b_b_ (488) > 1	−2.167	1.0495	0.2004	0.7917

As for CDOM, we found a significant covariance between CDOM on the b_b_ at 532 nm in which high CDOM (1–9 ppb) corresponds to decrease in b_b_ (532) by 15% (Fig 6c and Figs 7c, 8c, 9c) compared to the low CDOM level (0.1–1 ppb). In addition, increase in CDOM is observed with decrease b_b_ (440) by 5%. This could be explained by the strong absorption behavior of CDOM t blue relative to green wavelengths [36]. Thus, in theory, CDOM doesn’t show scattering behavior. This covariance has not been observed in the other wavelength (650 nm) in which an increase in CDOM has caused an insignificant increase in b_b_ (650) by 6% [37]. That being mentioned it is important to note that the scattering property of CDOM is always ignored, and the light absorbance of CDOM in the water is usually considered.

**Fig 11:**
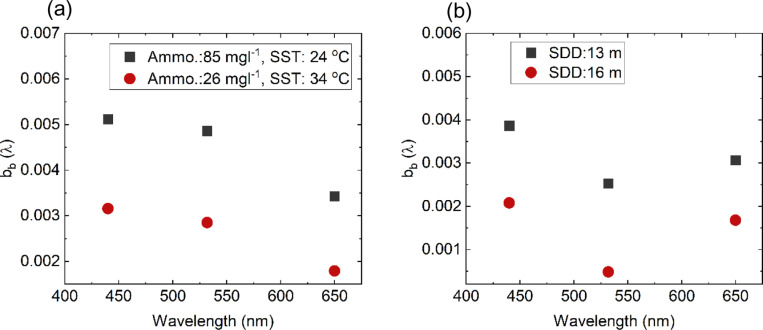
(a) b_b_(λ) for two different levels of Ammo. and SST while the other parameters are fixed including SDD:10m, Chl-*a*:0.35 mgm^-3^, Salinity: 40.5 g/l and (b) b_b_(λ) for two different levels of SDD while the other parameters are fixed including Chl-*a*:0.45 mg m^-3^, salinity:40 g/l and Ammo.:78 mg/l and SST:23 oC.

Ammonium (high level: 51 - 94 mg l^ − 1^ and low level: 11 - 50 mg l^ − 1^) is found to affect the b_b_ at the three wavelengths exceeding 47% with maximum effect on 650 nm followed by 440 nm (Fig 6d and Figs 7d, 8d, 9d). The effect is found to be significant but the least at 532 nm. Few studies have investigated the relationship between the backscattering and Ammonium (or Ammonia). Among these studies, Dong, Guoquan, et al. reported strong positive correlations between Ammonium and these wavelengths 490 nm, 560 nm, 665 nm, and 842 nm [38]. To support these findings, two cases of different levels of Ammonium and SST while the other parameters are fixed including SDD:10m, Chl-*a*: 0.35 mgm^-3^, Salinity: 40.5 g/l are examined as shown in Fig 11a. Based on this figure, it is clear that the increase in Ammonium has caused a significant increase in b_b_ at the three wavelengths.

#### 3.3.4 Variations of temperature and turbidity versus b_b_(λ).

According to the average vertical total backscattering measured against two SST categories, high (31 − 36 °C) and low (22 − 30 °C). There is insignificant change of b_b_ towards the increase in SST as shown in Fig 6e and Figs 7e, 8e, 9e. For example, the observed change of b_b_ (532) nm is found to be insignificant with a drop by 4%. To further support our findings, Fig 11a shows two cases of different levels of SST and Ammonium at fixed parameters mentioned in section 3.3.2. It is clear that b_b_(λ) decreases at 440, 532 and 650 nm with significant increase in SST by 10 °C. The resultant decrease in b_b_ is consistent to what was reported in [31] where low scattering was found at 26 °C compared to 20 °C at 546 nm.

As for the variation in water turbidity, the transparency of the water or turbidity has been measured here as the Secchi desk in which high SDD values indicate low turbidity (High SDD: 10–16 m) whereas low SDD showed high turbidity (Low SDD: 5–10 m). Our analysis showed a clear tendency between b_b_ at the three wavelengths and SDD where always low SDD has shown high b_b_ as shown in Fig 6f. This is significant for the three wavelengths where there is an increase with a range of 43–77% with the maximum increase at b_b_ (650) (Figs 7f, 8f, 9f). In addition, Fig 11b shows two cases of different SDD levels (13 m and 16 m) while the other parameters are fixed including Chl-*a*: 0.45 mg m^-3^, salinity: 40 g/l and Ammo.:78 mg/l and SST: 23 °C. As shown in the figure, the increase in SDD has shown a significant drop in b_b_ (λ) at the three wavelengths. This finding is consistent with the reported effect of the higher concentrations of suspended sediment on the 0.5–0.8 μm band signal [39].

Table 2 summarizes the general tendency of b_b_ (λ) according to the levels of the physical and chemical parameters. Increasing salinity, Ammonium, and turbidity are always associated with increasing b_b_ (λ). However, higher Chl-*a* and CDOM are associated mainly with lower b_b_ (λ) especially at the blue and green spectral range. In addition, the increase in the SST is associated with the decrease in b_b_ (λ).

**Table 2:** A summary of the general tendency of b_b_ (λ) according to the levels of the physical and chemical parameters including Ammo, Salinity, Chl-*a*, SDD, and CDOM. The light gray shows proportional relation between b_b_ (λ) and the parameters and dark gray shows the opposite.

b_b_ (440)		b_b_ (532)		b_b_ (650)	
Increase	Decrease	Increase	Decrease	Increase	Decrease
High Ammo.		High Ammo.		High Ammo.	
High Salinity		High Salinity		High Salinity	
	High Chl-*a*				High Chl-*a*
	High CDOM		High CDOM	High CDOM	
	High SDD		High SDD		High SDD

### Limitations

The Arabian Gulf experiences extreme environmental conditions, including high temperatures, salinity variations, and episodic events like dust storms and freshwater influx from rivers or desalination plants. Sampling at fixed stations may not adequately capture the rapid changes in water properties during such events, leading to incomplete or less accurate data. For example, heavy rainfall, though infrequent, can cause abrupt changes in salinity and turbidity, while dust storms may introduce fine particles that are not easily detected by the Secchi disk method. Additionally, the depth limitation of the ECO-triplet sensor might miss significant variations in water properties during vertical mixing events, which are common in the Gulf due to strong winds and shallow water.

This method can be applied to other coastal regions but might need adjustments for areas with more complex water dynamics, such as strong currents or significant organic matter pollution. In regions with abrupt changes, like heavy rain or pollution events, more frequent sampling or real-time monitoring would provide better coverage and improve the reliability of results.

## Conclusion

In hypersaline waters such as the Arabian Gulf, the relationship between backscattering coefficients and physical and biological properties is unclear, and the relationship may differ from that of other oceanic regions which contribute the majority of data used to develop optical models. The present study analyzes the covariation of salinity, temperature, chlorophyll-a, color dissolved organic matter (CDOM), ammonium, and turbidity of seawater backscattering. The results indicate that there is a possibility of the b_b_ (λ) decreasing by 10 at 440 and 23% at 650 nm by increasing Chl-*a*. The increase in salinity is associated with an increase in b_b_(λ) up to 19% which is within the previously reported range. In addition, the increase in turbidity and ammonium is associated with an increase in b_b_(λ) by 43–77% and > 47%, respectively. Thus, High ammonium, and high turbidity are always associated with high b_b_ (λ). In contrast, the increase in CDOM has been accompanied by a decrease in b_b_ (λ) especially at 532 nm. We found that the wavelength of 488 nm could be problematic especially for SST and salinity as there is an increase of b_b_ at this wavelength co-exist in both conditions which are hard to say if it is salinity or SST but could be a mixed covariance.

These findings have direct implications for the UAE economy. By clarifying how these factors affect optical properties, the research supports optimized practices in fisheries and aquaculture, leading to better management and sustainability. In coastal tourism, understanding these dynamics ensures high water quality, which is crucial for maintaining attractive and clean marine environments for visitors. For the oil and gas sector, the study’s insights help mitigate environmental impacts and ensure compliance with regulations, which is essential for sustainable operations. Thus, the research not only advances scientific knowledge but also provides valuable data that drives industry best practices, contributing to the UAE’s economic stability and resilience against climate change.
